# Amlamax™ in the Management of Dyslipidemia in Humans

**DOI:** 10.4103/0250-474X.44604

**Published:** 2008

**Authors:** B. Antony, B. Merina, V. Sheeba

**Affiliations:** R&D Laboratory, Arjuna Natural Extracts Ltd., P.B. No. 126, Bank Road, Alwaye-683 101, India; 1 Little Flower Hospital and Research Centre, Angamaly-683 572, India

**Keywords:** Amlamax, dyslipidemia, *Emblica officinalis*, ellagitannins

## Abstract

Hypercholesterolemia is the major cause of cardiovascular diseases leading to myocardial infarctions leading to considerable morbidity and mortality. During the past decade a group of molecules referred to as statins such as simvastatin, atrovastatin have been tried with great success in reducing total cholesterol. These molecules act by inhibiting the HMG CoA reductase enzyme thereby interfering with the synthesis of cholesterol. But statins reduce all the cholesterol including HDL cholesterol. Long term drug vigilance activity has revealed serious side effects of tendinopathy and related musculoskeletal disorders in some of the subjects. In an effort to manage hypercholesterolemia without serious side effects in a natural way we had tried the use of Amlamax™ a reconstituted, purified, standardized dried extract of *amla* (*Emblica officinalis*) containing 30% ellagitannins with other hydrolysable tannins on humans. We report the hitherto unobserved significant elevation of HDL cholesterol by the administration of Amlamax™

*Emblica officinalis* is widely used in many of the indigenous medical preparations against a variety of disease conditions^1^. Efficacy of *E. officinalis* in treating hypercholesterolemia and atherosclerosis is well established in animal experiments and human studies^2^–^4^. The pharmacological and clinical studies have indicated that *amla* has potent antioxidant effects against several test systems such as superoxide radicals, lipid peroxide formation induction by Fe^+++^/ADP ascorbate system, hydroxyl radical scavenging action and in systemic augmentation of antioxidant enzymes in the brain of laboratory animals^5^. A recent study conducted in rats found that flavonoids from *E. officinalis* effectively reduced lipid levels in serum and tissues and had a significant inhibitory effect of hepatic HMGCoA reductase activity^6^. The effect of standardized amla extract on atherosclerosis and dyslipidemia on animals is well studied^7^. The tannoid principles of fruits of *E. officinalis* have been traced to its antioxidant activity *in vitro* and *in vivo*^8^. A study conducted in rats found that emblicanin-A and emblicanin-B enriched fractions of fresh juice of emblica fruits showed antioxidant activity in ischemia-reperfusion-induced oxidative stress in rat heart^9^. The aqueous extract of *E. officinalis* fruit increased cardiac glycogen levels and decreased serum GOT, GPT and LDL in rats having induced myocardial necrosis^10^. Elevation of HDL is a remarkable result obtained in the present study, and a patent application for the composition is under processing^11^.

The main objective of this study is to explore the effectiveness of the herbal extract Amlamax™ in reducing dyslipidemia and elevation of HDL cholesterol, when administered along with dietary restriction and exercise. The present study investigated the effects of Amlamax™, a reconstituted standardized extract of *amla* containing 30% gallo ellagi tannins, with special emphasis on and confirmation of the elevation of HDL cholesterol, observed earlier in rabbits, and the safety and dose effectiveness of Amlamax™ for clinical treatment of hypercholesterolemic humans.

Fresh fruits of *amla* were collected from Coimbatore during August 2001 and pharmacognostically identified by comparing with voucher specimen number AE-HBRS-011. The chemicals used for the reactions were purchased from Merck. Fresh fruits of *amla* (*E. officinalis*) (100 kg) were cleaned, crushed, deseeded and refluxed with 50% methanol for 2 h. Then it was cooled and filtered. The filtrate was collected and the residue re-extracted with 50% methanol. The process was repeated twice and all the filtrates were pooled. The combined extracts were stripped of methanol under reduced pressure and subjected to membrane filtration (reverse osmosis) to obtain a concentrate of ca 40% TDS (total dissolved solids). The retentate was dried using ATFD (agitated thin film dryer) under controlled conditions, to obtain dried powdered extract containing ca 30% polyphenols of light greenish to dark greenish brown, free flowing slightly hygroscopic powder. The yield obtained was 4.2%. This hygroscopic extract was standardized to contain approximately 30% of total ellagitannins (emblicanin A and B) and kept at standardized conditions for further studies^12^. This dry extract in a free flowing powder form was filled in hard gelatin capsules (500 mg per capsule).

A two arm clinical trial with positive control was conducted at Little Flower Hospital and Research Centre, Angamaly, Kerala after the approval of ethical committee of the Hospital. A total of 68 patients attended the first visit. Dyslipidemic patients aged 35-45 years selected for the study drawn from the out-patient department of Little Flower Hospital, Angamaly, Kerala, India. Patient selection was based on the stratified random sampling principles and a total of 30 patients were selected for the study (15 Control and 15 Intervention groups). The patients suffering from chronic disorders (valvular heart disease, congestive cardiac failure, diabetes, gout, renal and thyroid disorders) and those consuming drugs interfering with lipid metabolism were excluded from the study. Those patients who were willing to participate in the study for 4 mo were selected. Selected patients were divided into intervention group (serum total cholesterol >240) and control group (serum total cholesterol: 200-240) after getting written consent from them. Fifteen patients were included in each group. Both intervention and control group were advised to follow a modified diet (low fat diet) and exercise. Beyond this intervention group were advised to take Amlamax™ 500 mg hard gelatin capsules *bid* after meals. Patients were advised to report monthly for check up and blood analysis. Patients were divided into two groups by randomisation as per standard statistical methods to minimize bias in the study. Group I- Amlamax™ was administered orally a 500 mg capsules (*bid*, intervention group, 15 subjects). Group II- Dietary restriction and exercise only (control group, 15 subjects)

All patients visited the clinic five times during the treatment period. During the first visit the informed consent from the patients were obtained. After getting the medical history, general examination was performed and lipid profile was checked (TC, HDL, LDL, VLDL and TG). Serum total cholesterol and HDL were estimated by enzymatic method^13^,^14^. Triglyceride was estimated by GPO-PAP method^15^. LDL and VLDL were calculated using Friedwald formula^16^.

During second visit i.e., the treatment initiation visit, pre-examination was done on screened patients. Baseline testing parameters like vital signs (systolic/diastolic pressure, pulse rates), haemogram (RBC, WBC, TC, DC, Hb, ESR) were analysed and started appropriate treatment as per randomised schedule. After starting the treatment, the patients were asked to visit the department for periodic check up such as BMI, vital signs, lipid profile and haemogram. After 4 mo treatment, liver function tests (LFT, serum bilirubin, AST, ALT and ALP) and renal function tests (blood urea, serum creatinine) were assessed. Data analysis was carried out by multifactorial analysis of variance (ANOVA).

The detailed evaluation of the vital signs recorded on both the control and the treatment groups showed no difference. All values measured for AST, ALT and serum bilirubin were within normal limits. This showed that Amlamax™ was non-toxic to the liver for the doses tested. The RFT parameters showed no significant differences at the end of the 4 mo study between the control and the intervention groups. All values of blood urea and serum creatinine were within clinically acceptable range for both groups, showing that Amlamax™ in the dose range did not cause any toxicity or load in the kidneys. No adverse reaction was noted on any of the subjects of the treatment group and general health was good. Thus it was established that Amlamax™ in the dose mentioned above is absolutely safe for clinical application.

The current treatment of choice for hypercholesterolemia is the use of statins. However, musculoskeletal side effects reported at least in certain cases of human subjects pointed out that measures have to be taken for cholesterol management with safer ways^17^. In an effort to manage hypercholesterolemia, Amlamax™, the reconstituted purified standardised dried extract of amla containing 30% galloellagitannins along with other hydrolysable tannins was administered on humans.

With respect to the clinical benefits of the treatment of hypercholesterolemic subjects receiving Amlamax™ 500 mg capsules *bid*, it was observed that there was a significant reduction of total serum cholesterol (17%), LDL cholesterol (21%) and triglycerides (24%) in addition to a remarkable, hither to unobserved elevation of HDL cholesterol by 14% (Table 1). Statistical analysis by ANOVA of the results confirmed the significance of the above observation with the reduction in LDL, VLDL and triglycerides (p<0.01) and HDL elevation (<0.05). In the control group there is a small (not significant) reduction in HDL cholesterol.

**TABLE 1:** LIPID PROFILE OF AMLAMAX™ GROUP AND CONTROL GROUP

Parameter	Amlamax™ group					Control group				
		
	0 mo	1 mo	2 mo	3 mo	4 mo	0 mo	1 mo	2 mo	3 mo	4 mo
TC (mg/dl)	281.9±10	266.5±8.5	251.7±10.6	234.5±11.6*	231±11.2*	235.5±18.3	234.6±16.5	234.4±13.5	241.4±12.8	238.0±12.1
HDL (mg/dl)	41.9±2.2	41.2±2.0	43.5±4.4	47.5±3.3**	48.0±2.9	43.1±3.7	41.1±2.3	42.6±3.9	41.3±3.4	42.0±3.5
LDL (mg/dl)	202.1±5.8	193.7±9.3	177.3±9.7*	159.1±13.2*	157±12.8*	170.8±18.2	168.6±16.3	163.3±11.6	172.0±11.6	171.0±11.4
VLDL (mg/dl)	40.63±4.4	40.45±4.8	36.0±4.03	31.27±4.6*	31.0±4.4*	21.63±1.4	24.88±2.0	29.0±3.5	28.13±2.6	29.0±2.8
TG (mg/dl)	208.7±19.6	194.5±24.3	182.2±17.6	159.2±21.8*	157±18.5*	107.6±6.9	117.4±8.0	145.9±17.7	141.4±12.5	143.0±12.4

This table shows the lipid profile of the intervention group (having total cholesterol>240) and control group (having total cholesterol-200-240).This study was for four months. Data’s were analysed using repeated measures of ANOVA. Values are represented mean±SE.

* significant decrease p<0.05.

** significant increase p<0.05

The reduction in serum cholesterols (LDL, VLDL and total cholesterol) by Amlamax™ may be explained by the inhibition of HMGCoA reductase enzyme thereby preventing the cholesterol synthesis^7^. Studies on animals conducted also ** support the inhibition of HMGCOA reductase by the intake of *amla* fruits as well as *amla* extract^6^,^7^. The increase in HDL cholesterol may probably due to the upregulation of enzymes responsible for the transfer of cholesterol from low density lipoprotein to high density lipoproteins. Earlier animal study has shown the prevention of further extension of atheromatous plaques and reversal of already formed lesions^7^. The second beneficial therapeutic effects of Amlamax™ is the significant elevation in RBC levels (P<0.05; Table 2) showing the effectiveness of the drug as vitalizer. Further the levels of lymphocytes also seemed to be enhanced indicating that the immunity may be enhanced. There are no earlier reports on the effect of amla extract on the HDL-C level except the animal study published by Antony *et al*. This extract is found to be non-toxic both acute and sub acute in experimental rats up to a dose of 2 g/kg for three months^18^. The results of this present study show elevation in the HDL cholesterol levels in the treatment groups after 4 mo intake of Amlamax™.

**TABLE 2:** HAEMOGRAM OF INTERVENTION AND CONTROL GROUP

Category	Amlamax™ group					Control group				
		
	0 mo	1 mo	2 mo	3 mo	4 mo	0 mo	1 mo	2 mo	3 mo	4 mo
RBC (mill/cumm)	4.7±0.1	4.7±0.1	4.8±0.1	4.9±0.1*	4.9±0.1*	4.8±0.2	4.8±0.1	4.9±0.1	4.8±0.1	4.8±0.1
WBC (mill/cumm)	6.7±0.5	6.2±0.4	6.6±0.3	6.8±0.3	6.7±0.3	6.6±0.5	6.0±0.3	6.4±0.1	6.6±0.1	6.6±0.1
Lymphocytes (%)	2.9±0.2	2.9±0.2	3.0±0.2	3.0±0.2*	3.0±0.2*	3.2±0.2	3.06±0.2	3.06±0.2	3.2±0.2	3.1±0.2
Hb (g/dl)	12.6±0.3	12.9±0.2	13.0±0.2	13.0±0.2	13.0±0.2	12.1±0.1	11.5±0.2	11.8±0.2	11.9±0.2	11.8±0.2

This table shows the haemogram of the control group and intervention group. The control group has serum cholesterol between 200-240 and the intervention group<240, before taking Amlamax. The study was for four months. Data’s were analysed using repeated measures of ANOVA. Values are represented mean±SE.

* significant decrease p<0.05.

Many studies have come to the conclusion that with a 1% decrease in total cholesterol levels, the incidence of coronary artery disease (CAD) decreases by 2%^19^. The present results indicate the potential of Amlamax^TM^ to treat dyslipidemia in hypercholesterolemic patients. Improvements in all the lipid parameters in the treatment group, namely, reduction in the total cholesterol, LDL cholesterol and triglycerides and more importantly, enhancement of the beneficial HDL cholesterol observed for the first time, supports this conclusion, although a larger study group would have allowed us to make firm conclusions. Nevertheless, the result of the present pilot study deserves attention because the enhancement in HDL cholesterol is more significant than those achieved with current drugs (e.g. statins). A study with direct comparison with statins is also planned. The present results may prompt other research groups to explore amla as a safe drug to treat dyslipidemia and atherosclerosis through well controlled randomised trials involving a larger study group.
